# Methyl 4-hy­droxy-2-isopropyl-1,1-dioxo-2*H*-1,2-benzothia­zine-3-carboxyl­ate

**DOI:** 10.1107/S1600536811024573

**Published:** 2011-06-25

**Authors:** Muhammad Nadeem Arshad, Islam Ullah Khan, Muhammad Zia-ur-Rehman, H. M. Rafique, K. Travis Holman

**Affiliations:** a X-ray Diffraction and Crystallography Laboratory, Department of Physics, School of Physical Sciences, University of the Punjab, Quaid-e-Azam Campus, Lahore 54590, Pakistan; b Materials Chemistry Laboratory, Department of Chemistry, GC University, Lahore 54000, Pakistan; c Applied Chemistry Research Center, PCSIR Laboratories Complex, Ferozpur Road, Lahore 54600, Pakistan; d Department of Chemistry, Georgetown University, 37th and Oth St NW, Washington DC 20057, USA

## Abstract

In the crystal structure of the title mol­ecule, C_13_H_15_NO_5_S, the S and N atoms of the thia­zine ring exihibit the maximum deviations from the least-squares plane of 0.3008 (6) and 0.3280 (7) Å, respectively. The ring therefore adopts a half chair conformation. The thia­zine ring is twisted by an angle of 13.29 (7)° with respect to the aromatic ring. The isopropyl substituent is oriented at a dihedral angle of 53.2 (12)° with respect to the thia­zine ring. An intra­molecular O—H⋯O hydrogen bond occurs. Inter­molecular hydrogen bonding is observed in the crystal structure.

## Related literature

For the synthetic procedure, see: Arshad *et al.* (2011 ▶). For the biological activity of related compounds, see: Lombardino *et al.* (1971 ▶); Vidal *et al.* (2006 ▶); Turck *et al.* (1996) ▶; Zia-ur-Rehman *et al.* (2006 ▶). For related structures, see: Arshad *et al.* (2008 ▶, 2009 ▶). For graph-set analysis, see Bernstein *et al.* (1995 ▶).
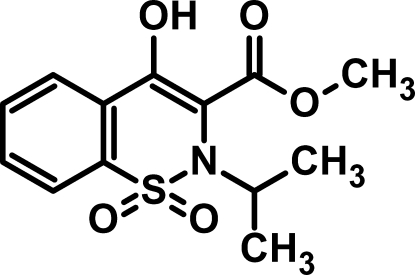


## Experimental

### 

#### Crystal data

C_13_H_15_NO_5_S
*M* *_r_* = 297.32
Monoclinic, 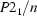

*a* = 11.3896 (6) Å
*b* = 9.8421 (5) Å
*c* = 12.7680 (7) Å
β = 105.782 (1)°
*V* = 1377.31 (13) Å^3^
*Z* = 4
Mo *K*α radiation
μ = 0.25 mm^−1^
*T* = 100 K
0.43 × 0.27 × 0.27 mm

#### Data collection

Siemens SMART 1K diffractometer with a Bruker APEXII detector
Absorption correction: multi-scan (*SADABS*; Bruker 2001 ▶) *T*_min_ = 0.899, *T*_max_ = 0.949
16137 measured reflections
3380 independent reflections
2967 reflections with *I* > 2σ(*I*)
*R*_int_ = 0.024

#### Refinement

*R*[*F* ^2^ > 2σ(*F* ^2^)] = 0.034
*wR*(*F* ^2^) = 0.092
*S* = 1.06
3380 reflections
187 parameters
H atoms treated by a mixture of independent and constrained refinement
Δρ_max_ = 0.43 e Å^−3^
Δρ_min_ = −0.40 e Å^−3^

### 

Data collection: *APEX2* (Bruker, 2001 ▶); cell refinement: *SAINT* (Bruker, 2001 ▶); data reduction: *SAINT*; program(s) used to solve structure: *SHELXS97* (Sheldrick, 2008 ▶); program(s) used to refine structure: *SHELXL97* (Sheldrick, 2008 ▶); molecular graphics: *ORTEP-3* (Farrugia, 1997 ▶) and *PLATON* (Spek, 2009 ▶); software used to prepare material for publication: *WinGX* (Farrugia, 1999 ▶) and *PLATON*.

## Supplementary Material

Crystal structure: contains datablock(s) I, global. DOI: 10.1107/S1600536811024573/im2299sup1.cif

Structure factors: contains datablock(s) I. DOI: 10.1107/S1600536811024573/im2299Isup2.hkl

Supplementary material file. DOI: 10.1107/S1600536811024573/im2299Isup3.cml

Additional supplementary materials:  crystallographic information; 3D view; checkCIF report

## Figures and Tables

**Table 1:** Hydrogen-bond geometry (Å, °)

*D*—H⋯*A*	*D*—H	H⋯*A*	*D*⋯*A*	*D*—H⋯*A*
C4—H4⋯O2^i^	0.93	2.49	3.311 (2)	147
C3—H3⋯O2^ii^	0.93	2.46	3.370 (2)	165
C11—H11⋯O1^iii^	0.98	2.39	3.317 (2)	157
O3—H3*O*⋯O4	0.88 (2)	1.77 (2)	2.578 (2)	151 (2)

Symmetry codes: (i) 
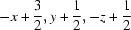
; (ii) 
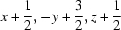
; (iii) 
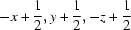
.

**Fig. 1.**
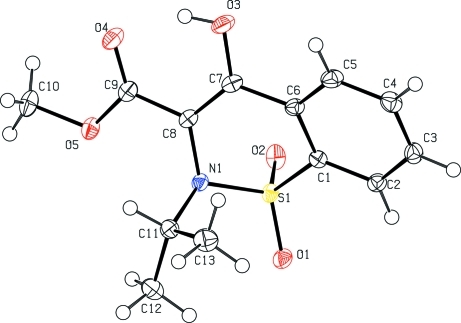
Ortep diagram for (I), thermal ellipsoids are drawn at the 50% probability level.

**Fig. 2.**
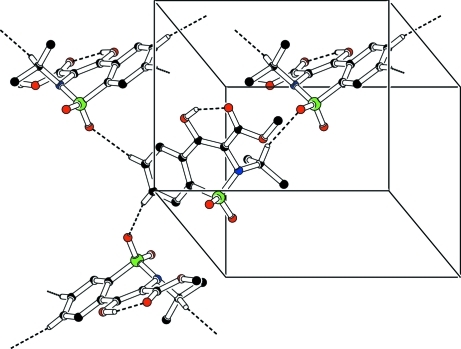
Unit cell packing for (I) showing hydrogen bonds as dashed lines. Hydrogen atoms not involved in hydrogen bonding interactions have been omitted.

## supplementary crystallographic information

### Comment

Benzothiazine 1,1-dioxide derivatives are the constituents of various drugs including Piroxicam and Meloxicam which are being used as non-steroidal anti-inflammatory drugs (NSAIDs) (Lombardino *et al.*, 1971; Turck *et al.*, 1996). Besides other biological activities (Zia-ur-Rehman *et al.*, 2006) these type of molecules have found their applications as intermediates (Vidal *et al.*, 2006). Our research group already reported the synthesis, biological activities (Arshad *et al.*, 2011) and crystal structures (Arshad *et al.*, 2008; 2009) of benzothiazine derivatives, II and III.

The title compound, I, is varied in structure with respect to III only concerning the alkyl group attached to the nitrogen atom of the thiazine ring. The characteristic intramolecular O—H···O hydrogen bond is observed in the structure of I as for II and III forming a six membered S^1^_1_(6) ring (C7/C8/C9/O4 H3O/O3) system (Bernstein, *et al.*, 1995). The observed ring is inclined at dihedral angles of 18.9 (4)° and 16.1 (4)° with respect to the thiazine (C1/C6/C7/C8/N1/S1) and aromatic (C1/C2/C3/C4/C5/C6) rings. The isopropyl group is oriented at a dihedral angle of 53.2 (1)° relative to the thiazine ring. The dihedral angle between the thiazine and aromatic ring is 13.29 (7)°. Alongwith the O—H···O type hydrogen bonding interaction the molecule is connected to it's neighboring symmetry equivalents by additional weak C—H···O type interactions producing a three dimensional network (Fig. 2. Tab. 1).

### Experimental

The synthesis of the titled compound has already been published (Arshad *et al.*, 2011). Recrystallization from methanol under slow evaporation of the solvent leads to the formation of crystals suitable for structural analysis.

### Refinement

Carbon bound H atoms were positioned geometrically with C—H = 0.93 Å and 0.98Å for aromatic and C11 carbon atoms, respectively, and were refined using a riding model with *U*_iso_(H) = 1.2 *U*_eq_(C). Similarly, H atoms of methyl groups were positioned geometrically with C—H = 0.96 Å and were refined using a riding model with *U*_iso_(H) = 1.5 *U*_eq_(C). The H atom of the hydroxyl group was located from the difference map with O–H= 0.88 (2)Å and was refined with *U*_iso_(H) = 1.5 *U*_eq_(O).

### Crystal data

**Table:**

C_13_H_15_NO_5_S	*F*(000) = 624
*M**_r_* = 297.32	*D*_x_ = 1.434 Mg m^−^^3^
Monoclinic, *P*2_1_/*n*	Mo *K*α radiation, λ = 0.71073 Å
Hall symbol: -P 2yn	Cell parameters from 7059 reflections
*a* = 11.3896 (6) Å	θ = 2.7–28.4°
*b* = 9.8421 (5) Å	µ = 0.25 mm^−^^1^
*c* = 12.7680 (7) Å	*T* = 100 K
β = 105.782 (1)°	Needle, colorless
*V* = 1377.31 (13) Å^3^	0.43 × 0.27 × 0.27 mm
*Z* = 4	

### Data collection

**Table:**

Siemens SMART 1K diffractometer with a Bruker APEXII detector	3380 independent reflections
Radiation source: fine-focus sealed tube	2967 reflections with *I* > 2σ(*I*)
graphite	*R*_int_ = 0.024
φ and ω scans	θ_max_ = 28.4°, θ_min_ = 2.1°
Absorption correction: multi-scan (*SADABS*; Bruker 2001)	*h* = −15→15
*T*_min_ = 0.899, *T*_max_ = 0.949	*k* = −13→12
16137 measured reflections	*l* = −16→16

### Refinement

**Table:**

Refinement on *F*^2^	Primary atom site location: structure-invariant direct methods
Least-squares matrix: full	Secondary atom site location: difference Fourier map
*R*[*F*^2^ > 2σ(*F*^2^)] = 0.034	Hydrogen site location: inferred from neighbouring sites
*wR*(*F*^2^) = 0.092	H atoms treated by a mixture of independent and constrained refinement
*S* = 1.06	*w* = 1/[σ^2^(*F*_o_^2^) + (0.0464*P*)^2^ + 0.6953*P*] where *P* = (*F*_o_^2^ + 2*F*_c_^2^)/3
3380 reflections	(Δ/σ)_max_ < 0.001
187 parameters	Δρ_max_ = 0.43 e Å^−^^3^
0 restraints	Δρ_min_ = −0.40 e Å^−^^3^

### Special details

**Table:**

Geometry. All s.u.'s (except the s.u. in the dihedral angle between two l.s. planes) are estimated using the full covariance matrix. The cell s.u.'s are taken into account individually in the estimation of s.u.'s in distances, angles and torsion angles; correlations between s.u.'s in cell parameters are only used when they are defined by crystal symmetry. An approximate (isotropic) treatment of cell s.u.'s is used for estimating s.u.'s involving l.s. planes.
Refinement. Refinement of F^2^ against ALL reflections. The weighted R-factor wR and goodness of fit S are based on F^2^, conventional R-factors R are based on F, with F set to zero for negative F^2^. The threshold expression of F^2^ > 2σ(F^2^) is used only for calculating R-factors(gt) etc. and is not relevant to the choice of reflections for refinement. R-factors based on F^2^ are statistically about twice as large as those based on F, and R- factors based on ALL data will be even larger.

### Fractional atomic coordinates and isotropic or equivalent isotropic displacement parameters (Å^2^)

**Table:**

	*x*	*y*	*z*	*U*_iso_*/*U*_eq_	
S1	0.45158 (3)	0.80155 (3)	0.24103 (3)	0.01449 (10)	
O1	0.41963 (9)	0.73289 (10)	0.32827 (8)	0.0204 (2)	
O2	0.46334 (9)	0.72358 (10)	0.14956 (8)	0.0208 (2)	
O4	0.32490 (9)	1.14730 (11)	−0.03579 (8)	0.0239 (2)	
O5	0.19108 (9)	1.00457 (10)	0.00818 (8)	0.0206 (2)	
O3	0.54813 (10)	1.14548 (11)	0.08196 (9)	0.0235 (2)	
N1	0.35223 (10)	0.92191 (11)	0.19471 (9)	0.0148 (2)	
C1	0.58852 (11)	0.89070 (13)	0.29337 (10)	0.0144 (2)	
C2	0.67716 (12)	0.84231 (14)	0.38274 (11)	0.0167 (3)	
H2	0.6646	0.7626	0.4174	0.020*	
C3	0.78531 (12)	0.91527 (15)	0.41972 (11)	0.0194 (3)	
H3	0.8463	0.8834	0.4788	0.023*	
C4	0.80243 (13)	1.03493 (15)	0.36896 (12)	0.0213 (3)	
H4	0.8745	1.0835	0.3950	0.026*	
C5	0.71319 (13)	1.08340 (15)	0.27951 (11)	0.0207 (3)	
H5	0.7258	1.1639	0.2459	0.025*	
C6	0.60455 (12)	1.01095 (14)	0.24018 (11)	0.0163 (3)	
C7	0.50866 (12)	1.05859 (14)	0.14559 (11)	0.0171 (3)	
C8	0.39091 (12)	1.01442 (13)	0.12351 (11)	0.0159 (3)	
C9	0.30108 (12)	1.06172 (14)	0.02519 (11)	0.0180 (3)	
C10	0.09884 (14)	1.05700 (17)	−0.08516 (12)	0.0271 (3)	
H10A	0.0828	1.1505	−0.0728	0.041*	
H10B	0.0252	1.0051	−0.0954	0.041*	
H10C	0.1276	1.0500	−0.1490	0.041*	
C11	0.28252 (12)	0.98889 (14)	0.26547 (11)	0.0181 (3)	
H11	0.2354	1.0621	0.2214	0.022*	
C13	0.36474 (14)	1.05728 (17)	0.36523 (12)	0.0258 (3)	
H13A	0.4031	0.9894	0.4173	0.039*	
H13B	0.3170	1.1166	0.3970	0.039*	
H13C	0.4262	1.1091	0.3444	0.039*	
C12	0.18920 (13)	0.89440 (17)	0.29256 (13)	0.0263 (3)	
H12A	0.1452	0.8473	0.2280	0.039*	
H12B	0.1332	0.9466	0.3205	0.039*	
H12C	0.2303	0.8297	0.3464	0.039*	
H3O	0.482 (2)	1.167 (2)	0.0300 (17)	0.039*	

### Atomic displacement parameters (Å^2^)

**Table:**

	*U*^11^	*U*^22^	*U*^33^	*U*^12^	*U*^13^	*U*^23^
S1	0.01430 (16)	0.01178 (16)	0.01607 (17)	0.00005 (11)	0.00187 (12)	0.00036 (11)
O1	0.0185 (5)	0.0178 (5)	0.0237 (5)	−0.0013 (4)	0.0035 (4)	0.0067 (4)
O2	0.0194 (5)	0.0180 (5)	0.0225 (5)	0.0020 (4)	0.0015 (4)	−0.0060 (4)
O4	0.0277 (5)	0.0229 (5)	0.0188 (5)	0.0008 (4)	0.0023 (4)	0.0069 (4)
O5	0.0186 (5)	0.0226 (5)	0.0176 (5)	0.0017 (4)	−0.0002 (4)	0.0023 (4)
O3	0.0259 (5)	0.0252 (5)	0.0178 (5)	−0.0055 (4)	0.0033 (4)	0.0078 (4)
N1	0.0158 (5)	0.0139 (5)	0.0145 (5)	0.0027 (4)	0.0041 (4)	0.0021 (4)
C1	0.0147 (6)	0.0149 (6)	0.0138 (6)	−0.0013 (5)	0.0043 (5)	−0.0027 (5)
C2	0.0189 (6)	0.0158 (6)	0.0157 (6)	0.0011 (5)	0.0055 (5)	0.0003 (5)
C3	0.0172 (6)	0.0237 (7)	0.0157 (6)	0.0019 (5)	0.0020 (5)	−0.0018 (5)
C4	0.0175 (6)	0.0252 (7)	0.0205 (7)	−0.0058 (5)	0.0042 (5)	−0.0036 (6)
C5	0.0225 (7)	0.0209 (7)	0.0190 (7)	−0.0056 (5)	0.0063 (5)	0.0019 (5)
C6	0.0179 (6)	0.0180 (6)	0.0134 (6)	−0.0011 (5)	0.0048 (5)	−0.0003 (5)
C7	0.0228 (7)	0.0153 (6)	0.0137 (6)	−0.0013 (5)	0.0054 (5)	0.0003 (5)
C8	0.0199 (6)	0.0142 (6)	0.0133 (6)	0.0006 (5)	0.0037 (5)	0.0007 (5)
C9	0.0214 (6)	0.0160 (6)	0.0161 (6)	0.0020 (5)	0.0040 (5)	−0.0008 (5)
C10	0.0232 (7)	0.0304 (8)	0.0220 (7)	0.0037 (6)	−0.0036 (6)	0.0038 (6)
C11	0.0191 (6)	0.0181 (6)	0.0182 (7)	0.0042 (5)	0.0072 (5)	0.0004 (5)
C13	0.0286 (8)	0.0290 (8)	0.0211 (7)	0.0014 (6)	0.0091 (6)	−0.0053 (6)
C12	0.0212 (7)	0.0291 (8)	0.0314 (8)	0.0017 (6)	0.0117 (6)	0.0037 (6)

### Geometric parameters (Å, °)

**Table:**

S1—O1	1.4319 (10)	C4—H4	0.9300
S1—O2	1.4338 (10)	C5—C6	1.3978 (19)
S1—N1	1.6338 (11)	C5—H5	0.9300
S1—C1	1.7556 (13)	C6—C7	1.4675 (18)
O4—C9	1.2264 (17)	C7—C8	1.3644 (19)
O5—C9	1.3361 (17)	C8—C9	1.4632 (18)
O5—C10	1.4524 (16)	C10—H10A	0.9600
O3—C7	1.3387 (16)	C10—H10B	0.9600
O3—H3O	0.88 (2)	C10—H10C	0.9600
N1—C8	1.4378 (17)	C11—C13	1.518 (2)
N1—C11	1.5072 (16)	C11—C12	1.521 (2)
C1—C2	1.3862 (18)	C11—H11	0.9800
C1—C6	1.4009 (18)	C13—H13A	0.9600
C2—C3	1.3926 (19)	C13—H13B	0.9600
C2—H2	0.9300	C13—H13C	0.9600
C3—C4	1.383 (2)	C12—H12A	0.9600
C3—H3	0.9300	C12—H12B	0.9600
C4—C5	1.390 (2)	C12—H12C	0.9600
			
O1—S1—O2	118.74 (6)	C8—C7—C6	122.54 (12)
O1—S1—N1	109.04 (6)	C7—C8—N1	121.61 (12)
O2—S1—N1	107.59 (6)	C7—C8—C9	119.64 (12)
O1—S1—C1	109.10 (6)	N1—C8—C9	118.75 (11)
O2—S1—C1	107.90 (6)	O4—C9—O5	123.06 (12)
N1—S1—C1	103.39 (6)	O4—C9—C8	122.64 (13)
C9—O5—C10	114.88 (11)	O5—C9—C8	114.29 (12)
C7—O3—H3O	104.6 (14)	O5—C10—H10A	109.5
C8—N1—C11	113.82 (10)	O5—C10—H10B	109.5
C8—N1—S1	112.76 (9)	H10A—C10—H10B	109.5
C11—N1—S1	121.74 (9)	O5—C10—H10C	109.5
C2—C1—C6	121.84 (12)	H10A—C10—H10C	109.5
C2—C1—S1	120.95 (10)	H10B—C10—H10C	109.5
C6—C1—S1	117.20 (10)	N1—C11—C13	113.06 (11)
C1—C2—C3	118.74 (13)	N1—C11—C12	112.55 (11)
C1—C2—H2	120.6	C13—C11—C12	112.96 (12)
C3—C2—H2	120.6	N1—C11—H11	105.8
C4—C3—C2	120.30 (13)	C13—C11—H11	105.8
C4—C3—H3	119.8	C12—C11—H11	105.8
C2—C3—H3	119.8	C11—C13—H13A	109.5
C3—C4—C5	120.81 (13)	C11—C13—H13B	109.5
C3—C4—H4	119.6	H13A—C13—H13B	109.5
C5—C4—H4	119.6	C11—C13—H13C	109.5
C4—C5—C6	119.86 (13)	H13A—C13—H13C	109.5
C4—C5—H5	120.1	H13B—C13—H13C	109.5
C6—C5—H5	120.1	C11—C12—H12A	109.5
C5—C6—C1	118.43 (12)	C11—C12—H12B	109.5
C5—C6—C7	121.37 (12)	H12A—C12—H12B	109.5
C1—C6—C7	120.19 (12)	C11—C12—H12C	109.5
O3—C7—C8	123.48 (12)	H12A—C12—H12C	109.5
O3—C7—C6	113.95 (12)	H12B—C12—H12C	109.5
			
O1—S1—N1—C8	167.45 (9)	C5—C6—C7—O3	20.73 (19)
O2—S1—N1—C8	−62.53 (10)	C1—C6—C7—O3	−159.37 (12)
C1—S1—N1—C8	51.48 (10)	C5—C6—C7—C8	−161.14 (13)
O1—S1—N1—C11	26.69 (11)	C1—C6—C7—C8	18.8 (2)
O2—S1—N1—C11	156.70 (10)	O3—C7—C8—N1	−179.43 (12)
C1—S1—N1—C11	−89.28 (11)	C6—C7—C8—N1	2.6 (2)
O1—S1—C1—C2	31.83 (13)	O3—C7—C8—C9	0.6 (2)
O2—S1—C1—C2	−98.46 (12)	C6—C7—C8—C9	−177.37 (12)
N1—S1—C1—C2	147.76 (11)	C11—N1—C8—C7	102.87 (14)
O1—S1—C1—C6	−149.00 (10)	S1—N1—C8—C7	−41.11 (16)
O2—S1—C1—C6	80.71 (11)	C11—N1—C8—C9	−77.14 (15)
N1—S1—C1—C6	−33.07 (11)	S1—N1—C8—C9	138.88 (11)
C6—C1—C2—C3	−0.5 (2)	C10—O5—C9—O4	−3.37 (19)
S1—C1—C2—C3	178.64 (10)	C10—O5—C9—C8	176.14 (12)
C1—C2—C3—C4	1.1 (2)	C7—C8—C9—O4	−4.9 (2)
C2—C3—C4—C5	−0.9 (2)	N1—C8—C9—O4	175.11 (12)
C3—C4—C5—C6	0.1 (2)	C7—C8—C9—O5	175.59 (12)
C4—C5—C6—C1	0.5 (2)	N1—C8—C9—O5	−4.40 (18)
C4—C5—C6—C7	−179.64 (13)	C8—N1—C11—C13	−80.86 (14)
C2—C1—C6—C5	−0.3 (2)	S1—N1—C11—C13	59.52 (15)
S1—C1—C6—C5	−179.45 (10)	C8—N1—C11—C12	149.66 (12)
C2—C1—C6—C7	179.81 (12)	S1—N1—C11—C12	−69.95 (14)
S1—C1—C6—C7	0.65 (17)		

### Hydrogen-bond geometry (Å, °)

**Table:**

*D*—H···*A*	*D*—H	H···*A*	*D*···*A*	*D*—H···*A*
C4—H4···O2^i^	0.93	2.49	3.311 (2)	147
C3—H3···O2^ii^	0.93	2.46	3.370 (2)	165
C11—H11···O1^iii^	0.98	2.39	3.317 (2)	157
O3—H3O···O4	0.88 (2)	1.77 (2)	2.578 (2)	151 (2)

Symmetry codes: (i) −*x*+3/2, *y*+1/2, −*z*+1/2; (ii) *x*+1/2, −*y*+3/2, *z*+1/2; (iii) −*x*+1/2, *y*+1/2, −*z*+1/2.
